# A Remarkable Depth Confusion in Images of the Incomplete Statues of Bruno Catalano

**DOI:** 10.1177/2041669519895990

**Published:** 2019-12-25

**Authors:** Rob van Lier, Vebjørn Ekroll

**Affiliations:** Donders Institute for Brain, Cognition and Behaviour, Radboud University, Nijmegen, The Netherlands; Department of Psychosocial Science, University of Bergen, Norway

**Keywords:** amodal completion, figure ground, illusory surfaces, knowledge-based completion

## Abstract

Images of Bruno Catalano’s sculptures of incomplete bodies give rise to a remarkable depth confusion in which the background is partly pushed to the front. We argue that this confusion is related to what happens in the Kanizsa square, although the effect in the images of Catalano’s sculpture appears to be driven by knowledge-based processing.

Bruno Catalano is a French sculptor who is famous for his incomplete body statues. When looking at two-dimensional images of these statues (Figure 1), something remarkable happens. The background that is seen through the gap in the body occasionally seems perceptually pushed to the front, such that it appears as a part of the body, or even as an occluding structure that conceals parts of a complete body from sight. In Figure 1, a few images are shown of Catalano’s statues. The depth confusion, however, is rather ambiguous and unstable. When observing the images, the different interpretations appear to compete with each other as the parts inside the body gaps can obviously also be seen as belonging to the background.^1^ This ambiguity in itself is of interest and adds to the aesthetic value of the incomplete statues, a phenomenon that relates to the effect of semantic instability in aesthetic appreciation (so-called SeIns, as put forward by Muth & Carbon, 2016).

**Figure 1.**
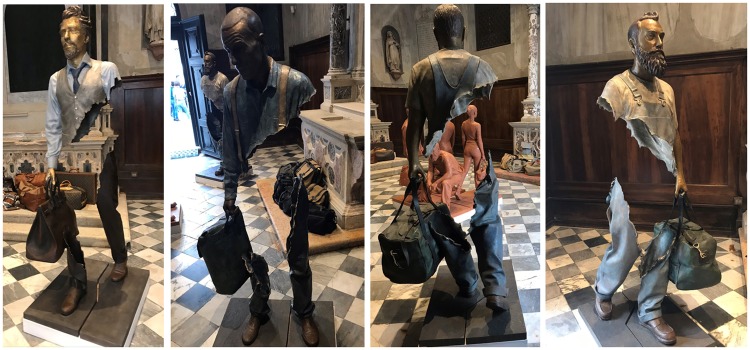
Various pictures of sculptures from Bruno Catalano’s series “Les Voyageurs.” Note how the background seen through the gap in the body is perceptually pushed to the front occasionally, such that it appears as a part of the body, or even as an occluding structure that conceals parts of the body from sight (all pictures in this figure are taken by Eva Specker at an exhibition in Venice, Italy, 2019). The reader is invited to search on the Internet for statues of Bruno Catalano to see more amazing examples (an often depicted statue with a strong confusing effect is “Le Grand van Gogh”).

The induced illusory depth order in the Catalano statues, although less stable, is related to what happens in the well-known Kanizsa square (Figure 2; Kanizsa, 1955, 1979), where the white inner square seems to be pushed forward, allowing an interpretation of four completed disks that are partly occluded by a white square. In Figure 2(a) and 2(b), examples are shown in which a simple background structure (the series of gray disks) is perceptually pushed forward (towards the observer). In Figure 2(a), the borders of two of the gray disks coincide with illusory borders of the illusory square such that the three disks in the middle appear to be part of the surface of the illusory square. This illusory percept relates to a phenomenon described by Ramachandran (1986), who showed that under specific conditions, a textured background can be “captured” by an illusory square (see also van Lier, de Wit, & Koning, 2006). Note that this kind of “capture” can also be experienced in Figure 1: When parts of the structure of the background align with parts of possible body contours, a confusing percept is evoked in which these background parts temporarily seems to belong to the bodies. In Figure 2(b), the gray disks cross the boundaries of the illusory square and seem to be pushed even further forward, as if there were four layers: First, closest to the observer, the gray disks, then the illusory square followed by four amodally completed disks, and finally, a white background (cf. van Lier et al., 2006). In contrast, Figure 2(c) provides an example in which the percept of an illusory square is completely abolished, as the inducing elements (the symmetrical crosses) no longer trigger completion processes (Kanizsa, 1955, 1979).

**Figure 2.**
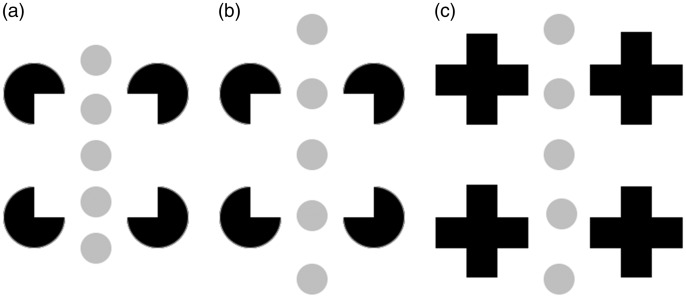
(a) A Kanizsa square with five gray disks in the middle. Note how the three middle disks appear in the same plane as the virtual white square. (b) Here, the five disks appear in front of everything else. (c) When the percept of the illusory square is abolished by changing the Pacman inducers into symmetrical crosses, the disks are not perceptually pushed forward.

In Catalano’s statues, the background is partly pushed to the front due to an apparent knowledge-driven completion of the body fragments. The strength of the effect depends on the recognizability of the fragments. To illustrate this aspect further, consider Figure 3(a). Notice that the picture triggers the confusing impression that the faces are partly occluded by a part of the background (or, stated otherwise, the background seems to act as an occluder, partially covering the faces). Figure 3(b) shows a manipulated version of Figure 3(a) in which the presence of faces is largely abolished. The rather abstract shapes in Figure 3(b) obviously have different properties (e.g., less details, different forms), but they cover approximately the same portion of the background and, importantly, have largely the same local contour properties at the gaps as in Figure 3(a) (i.e., gap length and angle of junctions at the gaps; similar to the Pacman-cross alteration in Figure 2). One may notice that in Figure 3(b), the depth confusion is much reduced. In Figure 3(c), the bodily features that are still present in Figure 3(b) were taken away further, which weakens the depth confusion even more.

**Figure 3.**
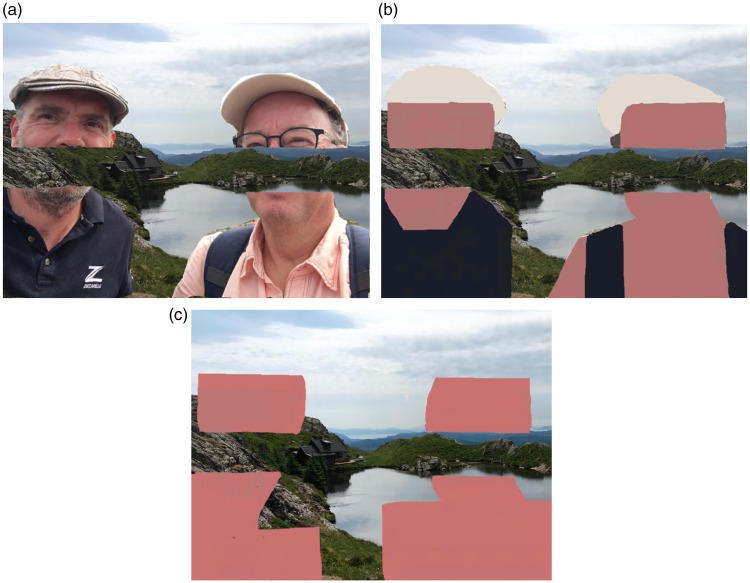
(a) A picture of the two authors with a large portion taken out such that the background is visible. Note that the impression is that part of the background is partly occluding the faces. (b) When the face and body parts are replaced by some more or less meaningless abstract shapes, while largely maintaining the local contour properties at the gaps, the illusory depth order is much less pronounced (i.e., the same area now is readily perceived as being part of the background). (c) The bodily features are removed further, leading to an even further reduction of the depth confusion.

A crucial difference between the above effect and the Kanizsa square is that while the alternative depth order in the Kanizsa square appears to be triggered predominantly by bottom-up processing, the depth order as seen in the example in Figure 3 and in Catalano’s sculptures seems to be driven by knowledge about what human faces and bodies look like. The phenomenon is similar to the perceived depth effects in some of Rene Magritte’s paintings (e.g., “Le Blanc-Seing”), in which, beside local figural occlusion cues, knowledge of biological shapes also appears to play a role (which in turn inspired Kanizsa to a similar composition in which a fragmented car was used; Kanizsa, 1985). It should be noted further that with regard to Catalano’s statues, the ambiguity is apparent in the static images, which would differ from observing the real three-dimensional statues as small head movements would quickly reveal the reality of the gaps.

All in all, the ambiguity when observing the incomplete statues of Bruno Catalano suggests that the body fragments temporarily trigger knowledge-based completions and with that cause the aesthetically pleasant depth confusions.

## Note

1 The confusion about the phenomenal impression on the Catalano statues was shared among many visitors at our poster presentation at *Visual Science of Art Conference* 2018, Trieste and the symposium *Skin of Things*, 2018 Amsterdam (van Lier & Ekroll, 2018), and also appears on various websites discussing Catalano’s statues.
